
JOURNAL INFORMATION
==============================
NLM Title Abbreviation: Function (Oxf)
Journal ID: Function (Oxf)
NLM Journal ID: 101770668
Journal ID: function

Function

EISSN: 2633-8823

ARTICLE INFORMATION
==============================
PMCID: PMC8903514
PMID: 35399494
DOI: 10.1093/function/zqac009
Article ID: zqac009
Article version: 1
Subjects: Perspectives, AcademicSubjects/SCI00960, AcademicSubjects/MED00772, AcademicSubjects/SCI01270, AcademicSubjects/SCI01360

Linking COVID-19 and Acute Pancreatitis Through the Pathogenic Effects of the SARS-CoV-2 S Protein Subunit 1 on Pancreatic Stellate Cells and Macrophages

Neoptolemos J P Department of General, Visceral and Transplantation Surgery, University of Heidelberg, Im Neuenheimer Feld 420, 69120 Heidelberg, Baden-Württemberg, Germany

Greenhalf W Liverpool GCPLab Facility, University of Liverpool, 1st Floor William Henry Duncan Building, 6 West Derby Street, Liverpool, L7 8TX, UK

Corresponding author. E-mail: John.Neoptolemos@med.uni-heidelberg.de
Electronic publication date: 2022 Feb 26
Electronic Location ID: zqac009
Copyright: © The Author(s) 2022. Published by Oxford University Press on behalf of American Physiological Society.
Copyright year: 2022
License: This is an Open Access article distributed under the terms of the Creative Commons Attribution License (https://creativecommons.org/licenses/by/4.0/), which permits unrestricted reuse, distribution, and reproduction in any medium, provided the original work is properly cited.
License URL: https://creativecommons.org/licenses/by/4.0/

Comment in: SARS-CoV-2 S Protein Subunit 1 Elicits Ca2+ Influx – Dependent Ca2+ Signals in Pancreatic Stellate Cells and Macrophages In Situ 3 2 31 1 2022 zqac002 Function 10.1093/function/zqac002 PMC8903325 35284826
Keywords: acute pancreatitis, SARS-CoV-2, COVID-19, calcium signalling, pancreatic stellate cells, macrophages
edited-state: accepted-manuscript
article-lifecycle: PAP

==============================

**PERSPECTIVES**

### Linking COVID-19 and acute pancreatitis through the pathogenic effects of the SARS-CoV-2 S protein subunit 1 on pancreatic stellate cells and macrophages

**Authors:**

[1]JP Neoptolemos and [2]W Greenhalf

[1]Department of General, Visceral and Transplantation Surgery, University of Heidelberg, Im Neuenheimer Feld 420, 69120 Heidelberg, Baden-Württemberg, Germany. John.Neoptolemos@med.uni-heidelberg.de

[2]Liverpool GCPLab Facility, University of Liverpool, 1st Floor William Henry Duncan Building, 6 West Derby Street, Liverpool, L7 8TX, UK. greenhaf@liverpool.ac.uk

**Corresponding author:**

Prof. Dr. med. John Neoptolemos, MA, MB, BChir, MD, FRCS, FMedSci, MAE

Professor of Surgery

Department of General, Visceral and Transplantation Surgery

University of Heidelberg

Im Neuenheimer Feld 420

69120 Heidelberg

Baden-Württemberg, Germany

John.Neoptolemos@med.uni-heidelberg.de

**Key words: acute pancreatitis, SARS-CoV-2, COVID-19, calcium signalling, pancreatic stellate cells, macrophages**

© The Author(s) 2022. Published by Oxford University Press on behalf of American Physiological Society. This is an Open Access article distributed under the terms of the Creative Commons Attribution License (http://creativecommons.org/licenses/by/4.0/), which permits unrestricted reuse, distribution, and reproduction in any medium, provided the original work is properly cited.

ORIGINAL UNEDITED MANUSCRIPT

**A Perspective on “SARS-CoV-2 S elicits Ca2+ influx-dependent Ca2+ signals in pancreatic stellate cells and macrophages in situ”.**

Since the early 1970’s Ole Peterson has been at the forefront of many of the major scientific discoveries that have moulded our understanding of pancreatic acinar cell physiology and of the pathophysiology leading to acinar cell necrosis and acute pancreatitis.[1] Pulse chase studies in the 1970s identified exocytosis as the key secretory pathway in pancreatic acini, occurring in response to physiologic concentrations of cholecystokinin or acetylcholine. This process involves the transport of proteins from the endoplasmic reticulum to the Golgi complex, concentration within condensing vacuoles and zymogen granules, and release into the extra-acinar lumen at the apical plasma membrane. Subsequent studies showed that supramaximal secretagogue stimulation resulted in blockade of apical exocytosis and release of intracellular granules from the baso-lateral plasma membrane into the intercellular space. This was shown to be a key pathogenic mechanism in the early evolutionary stages of acute pancreatitis. Normal exocytosis is governed by local cytosolic $Ca^{2+}$ spikes in the granular apical region. The $Ca^{2+}$ is released from intracellular stores in the endoplasmic reticulum via inositol trisphosphate and ryanodine receptors, and acid stores via the two-pore channel and ryanodine receptors. Apical cytosol $Ca^{2+}$ spiking also causes increased mitochondrial ATP production, to provide the energy source driving the mechanism of exocytosis.[1]

Pathogenic signals including fatty acids, alcohol, bile acids, and physical pressure result in sustained cytosolic $Ca^{2+}$ overloading. The consequences are direct trypsinogen activation within the zymogen granules with intra-cytosolic release of trypsin, as well as release into the intercellular space. There is also mitochondrial $Ca^{2+}$ overload with reduced ATP formation. These processes combine to produce acinar cell necrosis and an ever-expanding surrounding focus of tissue destruction. This is amplified by activation of both pancreatic stellate cells (by released kallikrein converted to bradykinin acting on the B2 receptor) generating toxic nitric

ORIGINAL UNEDITED MANUSCRIPT

oxide levels, and activation of resident macrophages (by released ADP and ATP acting on purinergic receptors) generating a series of cytokines including tumour necrosis factor-α. These processes result in further recruitment of circulating inflammatory cells into the foci of developing pancreatic necrosis. In clinically severe acute pancreatitis, the excessive inflammatory cell activation triggers an uncontrolled systemic anti-inflammatory response leading to multi-organ failure, especially affecting the lungs and kidneys.

In this latest work produced by Ole Petersen along with his longstanding co-workers Julia and Oleg Gerasimenko we are shown new insights into the pathogenic effects of the SARS-CoV-2 on pancreatic lobular cells.[2] The link between SARS-CoV-2 and pancreatitis was identified very early on in the COVID-19 pandemic but it's true significance remains unclear.[3] The nature of the link remains contentious, with some groups suggesting that the viral infection was not directly linked to the pancreatitis and even that many cases of pancreatitis were spurious since the raised amylase seen in patients with COVID-19 was mostly the result of a non-specific manifestation of shock in a critical illness. It has been open to debate whether this is direct viral damage of pancreatic cells or whether the damage is an indirect consequence resulting from the immune response.[4] This is of considerably more than academic interest, as it has been proposed that although diagnosis of acute pancreatitis is relatively rare in COVID-19, pancreatic inflammation may be a critical factor in COVID-19 associated deaths. In the context of a critically severe illness represented by COVID-19 the revised 2012 Atlanta Criteria for defining the diagnosis and level of severity may be misinterpreted and/or overinterpreted. This is illustrated by the COVID PAN collaborative study in which the results of serum amylase and/or lipase levels were not reported, and there was also no mention of the characteristic diagnostic findings of acute pancreatitis on contrast-enhanced computed tomography and magnetic resonance imaging.[5] Which leaves us with a

ORIGINAL UNEDITED MANUSCRIPT

pressing need to identify and characterise the specific mechanisms associated with the initiation of pancreatitis by SARS-CoV2 and how the severity is determined.

SARS-CoV-2 tropism towards distinct tissues is governed by cellular factors expressed on target cells. Viral entry requires attachment of the spike protein to angiotensin-converting enzyme 2 (ACE2).[6] The transmembrane serine protease 2 (TMPRSS2) is also required for cleavage of sites S1 and S2 for membrane fusion and receptor mediated endocytosis.[6] ACE2 and TMPRSS2 have both been identified in pancreatic endothelial pericytes and also in pancreatic ductal cells.[7,8] Expression of ACE2 and TMPRSS2 in human acini have also been found but this seems to be at low levels and patchy in distribution.[7,8] Genetic variants of TMPRSS2 may account for differential susceptibility to SARS-CoV-2 infection and COVID-19 disease severity.[9]

SARS- CoV-2 is also able to cause diffuse severe endothelial inflammation, and microvascular thrombosis in several anatomical sites, leading to diffuse microischaemia, an established cause of acute pancreatitis.[10] Nevertheless ischaemia can cause oedema of the pancreas along with raised circulating enzyme levels without necessarily causing pancreatitis, which requires acinar cell necrosis as a defining characteristic. Post-mortem examinations have detected SARS-CoV-2 nucleocapsid protein in pancreatic exocrine cells, raising the question whether infection of pancreatic ducts and acinar cells is associated not just with elevated amylase and lipase levels elevations in patients with SARS-CoV-2-associated pneumonia, but also acute oedematous pancreatitis.[8] None of the post-mortem studies on the four patients who died from COVID-19 however with pancreatic exocrine 2 nucleocapsid protein expression had any gross pathological or histological features of pancreatitis.[8]

Nevertheless, we can hypothesize three routes by which SARS-CoV-2 might cause acute pancreatitis: (1) direct infection of pancreatic acinar cells, (2) infection of pancreatic ductal cells with subsequent lobular disruption, and (3) microischaemia leading to pancreatic acinar

ORIGINAL UNEDITED MANUSCRIPT

necrosis. The paper by Gerasimenko and colleagues is a breakthrough in demonstrating that there is a direct action of SARS-CoV2 on the pancreatic parenchymal stellate cells leading to the production of IL-18 and activation of resident pancreatic macrophages.[2] Whether this is sufficient to initiate pancreatic acinar cell necrosis of itself is unclear at the present time, but may be contributory along with the aforementioned hypothetical mechanisms. Even so pancreatic IL-18 activated macrophages could promote clinical deterioration in otherwise critically ill patients. It is also significant that in the Gerasimenko model spike protein, or even just part of the spike protein, could cause pancreatitis in a susceptible individual, perhaps giving a mechanism to explain reports of acute pancreatitis following vaccination.

**Conflicts of Interest**

WG has no conflicts of interest; JPN holds the position of Editorial Board Member for Function and is blinded from reviewing or making decisions for the manuscript.

**References**

1. Petersen OH, Gerasimenko JV, Gerasimenko OV, Gryshchenko O, Peng S. The roles of calcium and ATP in the physiology and pathology of the exocrine pancreas. *Physiol Rev.* 2021;101(4):1691-1744. doi: 10.1152/physrev.00003.2021.
2. Julia V Gerasimenko, Ole H Petersen, Oleg V Gerasimenko. SARS-CoV-2 S Protein Subunit 1 Elicits Ca2+ Influx – Dependent Ca2+ Signals in Pancreatic Stellate Cells and Macrophages In Situ, *Function* 2022; 3: zqac002, https://doi.org/10.1093/function/zqac002
3. Wang F, Wang H, Fan J, Zhang Y, Wang H, Zhao Q. Pancreatic Injury Patterns in Patients With Coronavirus Disease 19 Pneumonia. *Gastroenterology*. 2020;159:367-70.

ORIGINAL UNEDITED MANUSCRIPT

4. de-Madaria E, Capurso G. COVID-19 and acute pancreatitis: examining the causality. *Nat Rev Gastroenterol Hepatol*. 2021;18: 3-4.

5. Pandanaboyana S, Moir J, Leeds JS, Oppong K, Kanwar A, Marzouk A, et al. SARS-CoV-2 infection in acute pancreatitis increases disease severity and 30-day mortality: COVID PAN collaborative study. *Gut* 2021; 70: 1061-1069. doi: 10.1136/gutjnl-2020-323364.

6. Hoffmann M, Kleine-Weber H, Schroeder S, Kr€uger N, Herrler T, Erichsen S, et al. SARS-CoV-2 cell entry depends on ACE2 and TMPRSS2 and is blocked by a clinically proven protease inhibitor. *Cell* 2020;181(2):271–80 16e8.

7. Coate KC, Cha J, Shrestha S, Wang W, Goncalves LM, Almaca J, et al. SARS-CoV-2 Cell Entry Factors ACE2 and TMPRSS2 Are Expressed in the Microvasculature and Ducts of Human Pancreas but Are Not Enriched in beta Cells. *Cell Metab.* 2020;32:1028-40 e4.

8. Müller JA, Groß R, Conzelmann C, Krüger J, Merle U, Steinhart J, et al. SARS-CoV-2 infects and replicates in cells of the human endocrine and exocrine pancreas. *Nat Metab.* 2021; 3:149-165. doi: 10.1038/s42255-021-00347-1.

9. David A, Parkinson N, Peacock TP, Pairo-Castineira E, Khanna T, Cobat A, Tenesa A, Sancho-Shimizu V, Casanova JL, Abel L, Barclay WS, Baillie JK, Sternberg MJ. A common TMPRSS2 variant has a protective effect against severe COVID-19. *Curr Res Transl Med.* 2022; 70:103333. doi: 10.1016/j.retram.2022.103333.

10. Pons S, Fodil S, Azoulay E, Zafrani L. The vascular endothelium: the cornerstone of organ dysfunction in severe SARS-CoV-2 infection. *Crit Care*. 2020; 24:353. doi: 10.1186/s13054-020-03062-7.

ORIGINAL UNEDITED MANUSCRIPT
